# End of life hospitalisations differ for older Australian women according to death trajectory: a longitudinal data linkage study

**DOI:** 10.1186/s12913-016-1729-3

**Published:** 2016-09-09

**Authors:** Melissa L. Harris, Xenia Dolja-Gore, Hal Kendig, Julie E. Byles

**Affiliations:** 1 Research Centre for Generational Health and Ageing, Faculty of Health and Medicine, University of Newcastle, University Drive, Callaghan, NSW 2308 Australia; 2 Centre for Research on Ageing, Health and Wellbeing, College of Medicine, Biology and Environment, Australian National University, Mills Road, Acton, ACT Australia; 3 ARC Centre of Excellence in Population Ageing Research, Sydney, NSW 2033 Australia

**Keywords:** Women, Old age, Hospital, Admissions, End of life, Chronic disease

## Abstract

**Background:**

Hospitalisations are the prime contributor to healthcare expenditure, with older adults often identified as high hospital users. Despite the apparent high use of hospitals at the end of life, limited evidence currently exists regarding reasons for hospitalisation. Understanding complex end of life care needs is required for future health care planning as the global population ages. This study aimed to investigate patterns of hospitalisation in the last year of life by cause of death (COD) as well as reasons for admission and short-term predictors of hospital use.

**Methods:**

Survey data from 1,205 decedents from the 1921–1926 cohort of the Australian Longitudinal Study on Women’s Health were matched with the state-based hospital records and the National Death Index. Hospital patterns based on COD were graphically summarised and multivariate logistic regression models examined the impact of short-term predictors of length of stay (LOS).

**Results:**

85 % of women had at least one admission in the last year of life; and 8 % had their first observed admission during this time. Reasons for hospitalisation, timing of admissions and LOS differed by COD. Women who died of cancer, diabetes and ‘other’ causes were admitted earlier than women who died of organ failure, dementia and influenza. Women who died of organ failure overall spent the longest time in hospital, and women with cancer had the highest median LOS. Longer LOS was associated with previous short- and medium-term- hospitalisations and type of hospital separation.

**Conclusions:**

Reducing acute care admissions and LOS at the end of life is complex and requires a shift in perceptions and treatment regarding end of life care and chronic disease management.

**Electronic supplementary material:**

The online version of this article (doi:10.1186/s12913-016-1729-3) contains supplementary material, which is available to authorized users.

## Background

Population ageing is expected to have widespread global implications for healthcare delivery and expenditure. In 2013, 841 million of the world’s population was aged over 60 years. It is projected that this figure will increase exponentially to two billion by 2050 [1]. In Australia, the proportion of adults aged over 65 has risen by approximately 30 % in the last decade. Ageing and health pressures are projected to result in a 24 % increase in government expenditure for health by 2055 [2]. This upward trend is projected across a number of other industrialised countries [3].

Hospitalisations have been identified as a prime contributor to healthcare expenditure, with older adults often identified as high hospital users [4, 5]. Proponents of the compression of morbidity hypothesis [6, 7] have focused on the disproportionate use of healthcare services towards the end of life, arguing that increased hospital use is associated with proximity to death rather than chronological age [8]. Menec and colleagues [9] found that 79 % of rural Canadian decedents were admitted to hospital at least once within the 6 months prior to death and around 60 % died while in hospital. Menec et al. [10] also found that 37 % of hospital use was concentrated into the last month prior to death, while an Australian study found a marked increase in admissions in the last 108 days for cancer-related deaths and 83 days for non-cancer related deaths [11].

Despite the apparent high use of hospitals at the end of life, examination of hospitalisation trajectories and reasons for hospitalisation using longitudinal population representative data across a number of causes of death is required. A recent retrospective chart study involving Dutch out-of-hours general practitioners (GPs) found that palliative care patients (identified by the terms such as palliative, terminal, and cancer) were most often hospitalised for the control of digestive, endocrine, metabolic and nutritional and respiratory symptoms [12]. Hospital admission was however dependent upon a number of factors including disease type, with cancer and cardiovascular cases more likely to be hospitalised. A follow-up GP-focused study comparing overnight hospitalisations in the 3 months prior to death for cancer and non-cancer patients found that while 71 different reasons were reported for the last hospitalisation, these differed according to death trajectory [13]. Cancer patients were most often admitted for the control of digestive symptoms and pain, while non-cancer patients were admitted for respiratory symptom control and cardiovascular complaints. Cancer patients were significantly more likely to have a shorter stay in hospital (<3 days) at their last admission and were less likely to die in hospital. Meanwhile, non-cancer patients were more likely to have multimorbidity and curative treatment as the goal prior to their last hospitalisation.

Previous cross-sectional and short-term follow-up studies have demonstrated that a number of patient-specific factors impact on the end of life care for older adults. In studies from the United States and Europe, age has been found to be associated with number of bed days and treatment decisions [10, 14]. Previous research has also demonstrated that functional capacity is a powerful predictor of high morbidity and mortality, particularly among hospitalised older adults [15, 16] and has been associated with end of life expenditure [17]. Kelley et al. [18] found that declines in functional capacity and increasing activities of daily living dependency were associated with increased number of bed days in the last 6 months of life. Lunney et al. [19] on the other hand found that functional decline fluctuates in the last year of life and is highly dependent of the type of disease trajectory, with marked differences noted for those with cancer, organ failure and frailty-related trajectories. When focused on death in hospital, increased associations have been found for living alone, presence of certain chronic diseases, ethnicity, rural residence, and fewer social contacts [9, 20, 21].

Improving end of life care is a global public health priority [22]. Understanding complex end of life care needs is required for future health care planning as the global population ages. However, previous research has largely focused on palliative care samples or specific community sub-populations, short observation periods and proxy recall. Understanding the reasons for hospitalisation and factors associated with hospitalisation requires examination of representative population level data across the spectrum of old age, including the very old (who are often excluded from research). This study aims to fill this gap by describing patterns of hospitalisation in the last year of life according to cause of death (COD) for older Australian women using data collected over a 12 year period. Reasons for hospitalisation and short-term predictors of hospital use are also explored.

## Method

### Overview of study design and participants

This study used data from the 1921–1926 cohort of the Australian Longitudinal Study on Women’s Health (ALSWH). Baseline surveys were completed in 1996 when the women were aged 70–75 years (*N* = 12,432). The cohort has been surveyed every 3 years since 1996. Participants were eligible for the study if they were New South Wales (NSW) residents and died between July, 2001 and June, 2013 (*N* = 1,205/4,363). Deaths records were obtained from the National Death Index (NDI) [23] and were matched on identifying information including name, address, gender, state, and date of birth.

### Measures

#### COD

Participants were classified into one of six groups based on primary COD in the NDI and using ICD-10 principle diagnosis codes. Similar to previous research [19, 24], the following conditions were included: cancer (ICD-10 codes C00-C997, D37, D44.0-D48.7), organ (i.e. heart, lung, kidney and liver) failure (ICD-10 codes I255.5, I42, I50-I51, J43-J44, J47, J61, J84, N18, K74), and Alzheimer’s disease/dementia (ICD-10 codes F03, R54, A81, F01, F03, G30). As diabetes (ICD-10 codes E10-E14) and influenza/pneumonia (ICD-10 codes J09-J18) also represent leading causes of death in this age group in Australia [25] and are associated with differing levels of disability [26], they were included as separate groups. All remaining causes of death were classified as ‘other’.

#### Hospital admissions

Hospital care in Australia is provided by a tax-funded universal healthcare system which receives funding at national and state levels and is managed separately by each state or territory. Data on admissions to hospital (bed days) in the 12 months prior to death (for each participant) were collected from the NSW Admitted Patients Data Collection (APDC). This database contains hospital inpatient information from public and private hospitals including admission and separation dates, and reason for admission (based on ICD-10 codes) [27].

#### Length of stay (LOS)

Women’s maximum admission length in the last year of life was categorised into one of three exclusive categories: (i) >0 and ≤5.8 days (acute LOS); (ii) >5.8 and ≤11.9 days (medium LOS); and (iii) >11.9 days (palliative LOS) based on the average length of stay for overnight separations and palliative admissions [28].

### Explanatory variables

Age at death was calculated from two strongly correlated datasets: the APDC (if the participant died in hospital) or alternatively the ALSWH dataset (using NDI data).

Baseline predisposing characteristics for each COD category were measured using data from the ALSWH 1996 survey. These included highest educational qualification, Country of birth, language spoken at home, smoking status [29], alcohol consumption and area of residence [30].

The following variables were included in the multivariate model examining short-term predictors of hospitalisation. Unless stated otherwise, variables were taken from the survey closest to the participant’s date of death. This included marital status, contributing to private health insurance, physical functioning and falls experienced in the previous 12 months. History of hypertension, arthritis and asthma were determined firstly from the COD ICD-10 codes. Missing data was filled in using either APDC hospital admission data (ICD-10 principle diagnoses) or ALSWH self-report (doctor diagnosis). Mode of separation was determined from the APDC and derived from the last hospital admission prior to death (home, death in hospital, nursing home).

### Ethical approval

The ALSWH project has ongoing ethical clearance from both the University of Newcastle and University of Queensland’s Human Research Ethics Committees. Ethical approval for the linkage of ALSWH survey data to the NSW APDC was received from the NSW Population and Health Services Research Ethics Committee, while linkage to the NDI was approved by the AIHW Ethics Committee. All participants provided informed consent for completion of the self-report survey and linkage to administrative data.

### Statistical analysis

Descriptive statistics were used to examine baseline and hospital characteristics for each of the COD groups. Patterns of hospital use over the 12 months prior to death and cumulative LOS days for each COD were graphically summarised. Three separate logistic regression models were constructed using backward elimination techniques to examine the impact of predisposing and health-related factors on LOS categories. Predictor variables entered into the multivariate analysis were chosen using a conservative significance level (*p* < 0.25). Due to the oversampling of participants from rural and remote areas, all models controlled for area of residence. The acute LOS model (comparing women categorised as having an ‘acute stay’ with those with no hospitalisation in the last year of life) also included age at death, time from last survey, mode of separation at last admission, marital status, contribution to private health insurance, COD, chronic conditions, physical functioning and falls. The medium LOS model (comparing women categorised as having a ‘medium stay’ to those women included in the acute LOS analysis [i.e. acute LOS and no hospitalisation]) included all variables from the acute LOS model with addition of number of acute length hospitalisations. The palliative LOS model (comparing women categorised as having a ‘palliative stay’ to those women included in the medium LOS analysis [i.e. medium LOS, acute LOS and no hospitalisation]) included all variables from the medium LOS model with the addition of number of medium length hospitalisations. The Hosmer and Lemeshow goodness-of-fit test was used to assess the fit for each model, with odds ratios (95 % Confidence Interval). Participants’ pre- and post-survey information was used to fill in missing data for predictor variables. The logistic regression analyses were performed using STATA v13 (Stata Corporation, College Station, TX). All other analyses were performed using SAS v9.4 (x64).

## Results

### COD profile

Among the 1,205 women who died during the observation period, 30.5 % (*n* = 382) of deaths were attributed to cancer, 21.7 % (*n* = 270) to organ failure, 13.1 % (*n* = 138) to dementia/Alzheimer’s disease, 7.0 % (*n* = 81) to diabetes and 3.7 % (*n* = 51) to influenza/pneumonia. The remaining 24.0 % (*n* = 283) were attributed to other causes such as acute myocardial infarction (I21.9), stroke (I64), other chronic heart and cerebrovascular disease (I25.9 and I69.8) and intracerebral hemorrhage (I61.9).

### Baseline characteristics according to COD

Baseline characteristics according to each COD ICD code are shown in Table 1. Compared to the other groups, a greater proportion of women died before the age of 80 when the COD was related to diabetes. Women with dementia/Alzheimer’s disease as the primary COD were most likely to have lived into very old age (44.5 %). In addition to age at death, predisposing baseline characteristics, smoking status, language spoken at home, and alcohol consumption were significantly associated with COD. Women who died of cancer were less likely to speak English as a second language (69.0 %) and more likely to be current smokers (13.3 %) compared to women in the other groups (with the exception of influenza). Women who died of diabetes-related causes were more likely to be non-drinkers (56.2 %), while those with influenza were more likely to be low risk drinkers (65.8 %) compared to other women.

**Table 1:** Baseline characteristics by cause of death for women from the 1921–1926 cohort residing in NSW who died during the period July 2001 and June 2013

	Other N (%)	Cancer N (%)	Organ Failure N (%)	Dementia/Alzheimer’s N (%)	Diabetes N (%)	Influenza/Pneumonia N (%)	*p*-value
Area of residence							
Metropolitan	117 (69.5)	140 (65.8)	98 (65.1)	68 (74.3)	35 (71.7)	15 (58.1)	0.238
Inner regional	120 (22.5)	180 (24.1)	116 (24.1)	49 (18.4)	27 (17.0)	31 (36.5)	
Outer regional/remote/very remote	46 (8.0)	62 (10.8)	56 (10.8)	21 (7.3)	19 (11.2)	5 (5.4)	
Predisposing factors							
^a^Average age of death	82.2 (3.5)	82.6 (3.4)	83.2 (3.7)	84.5 (3.2)	85.5 (3.9)	82.6 (3.5)	
Age at death							
Up to 79	87 (30.8)	94 (24.8)	60 (22.8)	13 (9.6)*	26 (32.7)	13 (28.1)	<0.0001
80–84 years	122 (40.3)	190 (49.5)	115 (43.1)	61 (45.9)	29 (36.2)	24 (45.6)	
85+ years	74 (29.0)	98 (25.6)	95 (34.2)	64 (44.5)*	26 (31.2)	14 (26.4)	
Highest qualifications							
No formal/year 10	212 (78.3)	284 (77.2)	199 (75.4)	102 (79.0)	67 (88.6)	36 (72.6)	0.223
Post school	49 (21.7)	78 (22.8)	51 (24.6)	8 (21.0)	8 (11.4)	13 (27.4)	
Country of birth	214 (71.9)	280 (68.4)	210 (72.6)	112 (79.2)	64 (78.4)	40 (74.1)	0.142
English as a second language	199 (76.0)	264 (69.0)	197 (74.0)	104 (81.5)	60 (81.3)	37 (78.1)	0.039
Smoking status							
Non-smoker	156 (56.6)	205 (59.0)	128 (53.0)	73 (55.3)	54 (71.8)	29 (57.3)	0.032
Ex-smoker	82 (34.5)	109 (27.7)	85 (34.2)	46 (36.3)	16 (25.9)	12 (25.7)	
Current smoker	25 (8.9)	49 (13.3)	38 (12.9)	10 (8.4)	1 (2.4)	7 (17.0)	
Alcohol consumption							
Non-drinker	98 (37.7)	125 (38.4)	95 (36.8)	44 (35.2)	37 (56.2)*	15 (30.9)	0.094
Low risk drinker	153 (58.8)	209 (56.7)	142 (58.2)	78 (57.2)	30 (42.5)	31 (65.8)*	
Medium/risky drinker	9 (3.5)	20 (5.0)	15 (5.0)	8 (7.6)	2 (1.3)	3 (3.3)	

^a^means and standard deviations are reported

*Greatest cell contribution to the χ2-test

### Hospital use in the last year of life

In all, 85 % of women had at least one hospital admission in the 12 months prior to death; and 8 % had their first observed admission during this final 12 months. There were significant differences between the 15 % of women not hospitalised compared to the 85 % that had at least one hospitalisation in the last year of life. Women who did not have a hospitalisation were less likely to be widowed (49 % vs 54.7 %), more likely to have died of causes other than the five conditions of interest (37 % vs 21 %) and more likely to have private health insurance (100 % vs 55 %).

The top ten medical reasons for hospitalisation (based on Australian Refined Diagnosis Related Groups) differed according to COD (see Additional file 1: Table S1). Although all groups of women were admitted at least once for rehabilitative purposes during this period, the greatest proportion of rehabilitation admissions were for women who died of influenza/pneumonia (15.7 %), organ failure (12.6 %) and ‘other’ causes (12.0 %). Women with influenza/pneumonia as their primary COD were also likely to be admitted for respiratory infection and inflammation (33.3 %), stroke and other cerebrovascular disorders (17.7 %) and other factors influencing health status (13.7 %). In addition to rehabilitative admissions, women who died of dementia/Alzheimer’s disease or ‘other’ causes were also likely to be admitted for stroke and other cerebrovascular disorders (10.1 % and 9.1 % respectively). Women with cancer as their primary COD were most often admitted for digestive disorders (7.3 %), respiratory infections (6.5 %) and post-surgery follow-up care (6.3 %). Women who died of organ failure had the highest proportion of admissions for chronic obstructive airway disorders (18.5 %) of all groups and were commonly admitted for heart failure and shock (16.7 %). Women who died of diabetes had the highest proportion of admissions for heart failure and shock (18.5 %) and circulatory disorders (18.5 %).

**Fig. 1:**
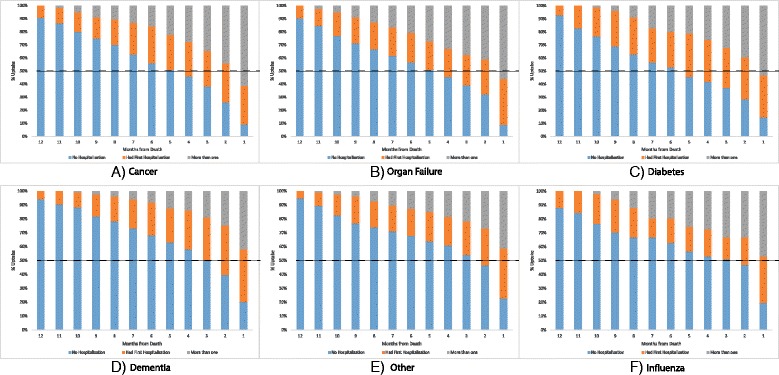
Timing of overnight hospital admissions in the 12 months prior to death for women from the 1921–1926 cohort for the six specific causes of death. The dashed line shows when 50 % of women for each specific cause of death had been admitted to hospital at least once in the last year of life

Timing of admission to hospital in the last year of life is shown in Fig. 1. Using the proportion at which 50 % of women had been admitted at least once as an indicator, women who died of cancer, diabetes and ‘other’ causes were admitted earlier with 50 % of women in these groups admitted by the fifth month prior to death. In contrast, women who died of organ failure, dementia and influenza/pneumonia took until the second and third month prior to death to reach the 50 % admission rate. As shown in Table 2, a similar pattern was found in terms of hospital use and proximity to death, with 71.4 % of women with influenza/pneumonia, and 63.6 % of women with diabetes dying within 1 month of their last admission, respectively.

**Table 2:** Hospital characteristics by cause of death for women from the 1921–1926 cohort

	Other N (%)	Cancer N (%)	Organ Failure N (%)	Dementia/Alzheimer’s N (%)	Diabetes N (%)	Influenza/Pneumonia N (%)	*p*-value
Death in hospital							
Yes	138 (49.5)	223 (59.4)	162 (59.2)	44 (29.5)	37 (44.9)	28 (55.5)	<0.0001
No	81 (27.6)	123 (31.0)	84 (32.4)	67 (51.8)	32 (37.9)	13 (25.3)	
Unknown^a^	64 (22.9)	36 (9.6)	24 (8.4)	27 (18.7)	12 (17.2)	10 (19.2)	
Independent living^b^	92 (33.3)	121 (32.1)	85 (31.6)	44 (32.1)	29 (36.3)	19 (38.0)	0.928
Days from last admission to death							
< 1 week	95 (34.7)	132 (32.3)	102 (34.5)	27 (19.5)	24 (31.5)	21 (39.5)	<0.0001
≥1 week <1 month	67 (22.2)	113 (29.3)	79 (28.7)	42 (32.1)	20 (32.1)	14 (31.9)	
≥ 1 month <3 months	31 (11.5)	63 (19.3)	24 (10.7)	19 (14.6)	11 (11.1)	2 (2.2)	
≥ 3 months < 1 year	26 (8.7)	38 (9.5)	41 (17.7)	23 (15.1)	14 (18.4)	4 (7.1)	
No admissions	64 (22.9)	36 (9.6)	24 (8.4)	27 (18.7)	12 (17.2)	10 (19.2)	
Hospital stay							
No hospitalisation	10 (19.6)	36 (9.4)^f^	24 (8.9)^f^	27 (19.6)	12 (14.8)	64 (22.6)^f^	<0.001
^c^Acute	10 (19.6)	58 (15.2)	51 (18.9)	30 (21.7)	18 (22.2)	56 (19.8)	
^d^Medium	9 (17.7)	80 (20.9)	62 (23.0)	33 (23.9)	14 (17.3)	50 (17.7)	
^e^Palliative	22 (43.1)	208 (54.5)	133 (49.3)	48 (34.8)	37 (45.7)	113 (39.9)	

^a^not hospitalised

^b^measured from the ALSWH survey closest to death

^c^an acute LOS was defined as >0 and ≤5.8 days

^d^a medium LOS was defined as >5.8 and ≤11.9 days

^e^a palliative LOS was defined as >11.9 days

^f^Greatest cell contribution to the *χ* ^2^-test

**Fig. 2:**
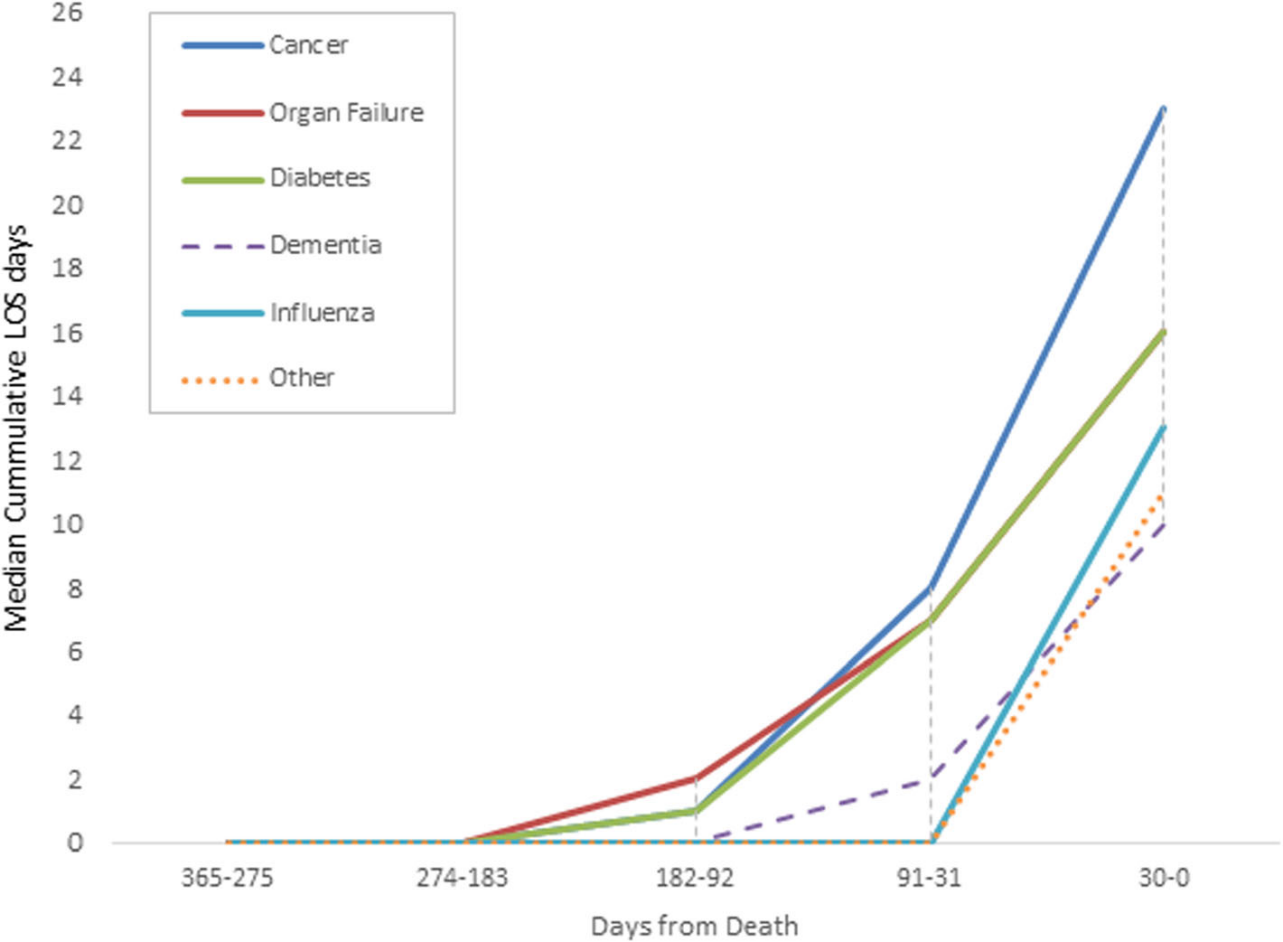
Median cumulative overnight length of stay days in the 12 months prior to death for women from the 1921–1926 cohort

The median cumulative LOS days for women in their last year of life according to their primary COD is shown in Fig. 2. No significant difference was found across the groups in terms of days in hospital within 274–365 days prior to death. Women who died of cancer had the highest median number of bed days (Median = 23), followed by diabetes (Median = 16), organ failure (Median = 16), influenza/pneumonia (Median = 13), ‘other’ (Median = 11) and dementia/Alzheimer’s disease (Median = 10). The greatest increase in average cumulative LOS days was seen within the last 30 days prior to death for women with a primary COD of cancer (13.9 days) compared to women with organ failure (11.2 days) or influenza (10.1 days). All other COD categories had an average increased stay of less than 10 days within the last 30 days prior to death. Differences were found for COD groups within 1 month of death (Kruskal-Wallis test = 38.4, *p*-value <0.0001).

Death in hospital status, ability to live independently and the days from last admission to death are shown in Table 2. Importantly, around 60 % of women who died of cancer and organ failure died in hospital, while 71.4 % of women with influenza/pneumonia, and 63.6 % of women with diabetes died within 1 month of their last admission.

### Predictors of acute, medium and palliative lengths of stay

The proportion of women in each of the COD categories who had a maximum LOS that was classified as either acute, medium or palliative, as well as those who were not admitted to hospital in their last year of life are also shown in Table 2. Women who died of influenza/pneumonia were less likely to be admitted to hospital in the last year of life. Women who died of diabetes (22.2 %) were more likely to be admitted for an acute LOS, while those with dementia were more likely to be admitted for a medium level LOS (23.9 %). Women who died of cancer (54.5 %) and organ failure (49.3) were more likely to have been admitted for a palliative level LOS.

Multivariable predictors of LOS are shown in Table 3. The final reduced acute LOS model shows that women who had been diagnosed or treated for arthritis were 72 % more likely to be hospitalised for an acute stay than women without arthritis (95 % CI:1.08,2.72). When COD was regressed onto LOS, women who died of dementia/Alzheimer’s disease and ‘other’ causes were approximately 40 % less likely than women who died of cancer to be admitted for an acute LOS.

**Table 3:** Predictors of acute, medium and palliative level length of stay (LOS) for women from the 1921–1926 cohort

Predictors	Acute LOS^a^ *N* = 396		Medium LOS^c^ *N* = 644		Palliative LOS^e^ *N* = 1,205	
	Full model OR (95 % CI)	Reduced model^b^OR (95 % CI)	Full model OR (95 % CI)	Reduced model^d^OR (95 % CI)	Full model OR (95 % CI)	Reduced model^f^OR (95 % CI)
Age at death	1.11 (1.05, 1.18)*	1.11 (1.04, 1.17)*	1.04 (0.99, 1.10)			
Time from last survey to death (days)			1.00 (1.00, 1.00)		1.00 (0.99, 1.00)	
Mode of separation from last admission						
Home			Reference	Reference	Reference	Reference
Died in hospital			3.99 (2.70, 5.92)*	4.16 (2.87, 6.02)*	2.09 (1.57, 2.78)*	2.76 (2.11, 3.62)*
Nursing home			2.83 (1.43, 5.58)*	3.07 (1.57, 6.00)*	3.04 (1.93, 4.79)*	3.86 (2.49, 5.99)*
^g^Widowed marital status	0.79 (0.52, 1.22)				1.16 (0.90, 1.51)	1.21 (0.95, 1.55)
^g^Private health insurance					0.93 (0.71, 1.21)	
^h^Number of acute level hospital admission			0.99 (0.85, 1.16)		0.98 (0.89, 1.08)	
^i^Number of medium level hospital admissions					1.16 (1.01, 1.34)*	1.27 (1.10, 1.45)*
Cause of death						
Cancer	Reference	Reference	Reference	Reference	Reference	Reference
Other	0.58 (0.33, 1.03)	0.60 (0.34, 1.06)*	0.54 (0.33, 0.88)*	0.53 (0.33, 0.86)*	0.66 (0.47, 0.94)*	0.61 (0.44, 0.86)*
Organ failure	1.15 (0.59, 2.24)	1.18 (0.61, 2.29)	0.81 (0.49, 1.33)	0.83 (0.51, 1.37)	0.83 (0.58, 1.18)	0.76 (0.54, 1.06)
Dementia/Alzheimer’s disease	0.60 (0.29, 1.21)	0.62 (0.31, 1.25)*	0.80 (0.44, 1.45)	0.82 (0.46, 1.45)	0.50 (0.31, 0.79)*	0.49 (0.32, 0.76)*
Diabetes	0.79 (0.32, 1.97)	0.81 (0.32, 2.00)	0.59 (0.27, 1.30)	0.63 (0.29, 1.35)	0.80 (0.46, 1.40)	0.80 (0.48, 1.33)
Influenza/pneumonia	0.61 (0.22, 1.70)	0.63 (0.23, 1.79)	0.45 (0.18, 1.13)	0.48 (0.19, 1.19)	0.68 (0.35, 1.33)	0.67 (0.35, 1.29)
^g^Hypertension	1.51 (0.98, 2.34)	1.50 (0.97, 2.31)	1.26 (0.88, 1.80)	1.35 (0.95, 1.91)	1.15 (0.89, 1.49)	
^g^Arthritis	1.74 (1.09, 2.76)*	1.72 (1.08, 2.72)*	1.13 (0.77, 1.65)			
^g^Asthma			1.99 (1.11, 3.58)*	2.00 (1.13, 3.53)*	1.30 (0.89, 1.49)	1.38 (0.94, 2.02)
Cardiovascular disease						
^g^Falls experienced in the previous 12 months	1.09 (0.89, 1.50)				1.14 (0.87, 1.49)	1.21 (0.94, 1.55)
Physical functioning				0.99 (0.99, 1.00)	1.00 (1.00, 1.00)	

^a^The cut point for acute level LOS was ≤5.8 days. Women with a maximum LOS of ≤5.8 days in their last year of life were compared with women who add no hospitalisations. A total of 223 (56.3 %) of this sample experienced an acute level LOS

^b^Hosmer-Lemeshow goodness of fit *p* value for the original model was 0.78 and 0.48 for the final model

^c^The cut points for a medium level LOS was >5.8 days and ≤11.9 days. Women with a maximum LOS was >5.8 days and ≤11.9 days in their last year of life were compared with women included in the acute level LOS analysis (i.e. those who had a maximum stay of ≤5.8 days (acute) or no hospitalisation). A total of 248 (38.5 %) of women of this sample experienced a medium level LOS

^d^Hosmer-Lemeshow goodness of fit *p* value for the original model was 0.14 and 0.51 for the final model

^e^The cut point for a palliative level LOS was >11.9 days. Women with a maximum LOS in their final year of life of >11.9 days were compared with women included in the medium LOS analysis (i.e. those who had a maximum LOS of >5.8 days and ≤11.9 days (medium), >0 and ≤5.8 days (acute) and no hospitalisation). A total of 561 (46.6 %) of women of this sample experienced a palliative level LOS

^f^Hosmer-Lemshow goodness of fit *p* value for the original model was 0.08 and 0.07 for the final model

^g^No is the reference category

^h^Only included in the medium and palliative LOS models

^i^Only included in the palliative LOS model

*Statistically significant (*P* <0.05)

When medium levels of stay were examined, significant associations were found for mode of separation, number of acute hospitalisations and COD. Importantly, a two-fold increase in risk of having a medium level LOS was noted for women with asthma (95 % CI:1.13,3.53) compared to those without the condition, while a 3–4-fold increase in risk was found for those who died in hospital (OR = 4.16, 95CI:2.87,6.02) or were discharged to a nursing home (OR = 3.07, 95 % CI:1.57,6.00) compared to women who were discharged to home. Compared to women who died of cancer, a 47 % (95%CI:0.33,0.86) decrease in risk of a medium level LOS was noted for those who died of ‘other’ causes. Similarly, mode of separation, number of medium level hospitalisations and COD were associated with a palliative level LOS.

## Discussion

Although examination of hospital use among older adults has been a key focus due to its relevance to policy and future healthcare planning, hospitalisation during the end of life period has received less attention, with much of the focus on healthcare expenditure [31, 32]. Using 12 years of nationally representative data we were able to demonstrate that for older women [33], hospital use in the last year of life is complex and differs according to COD. Women who died of organ failure spent the longest time in hospital over the observation period, while women with cancer had the highest median LOS. Longer lengths of stay (medium and palliative levels) were found to be primarily driven by mode of separation and number of shorter-term admissions. The findings of this study have important implications for healthcare pathways at the end of life, including improving the access to, and integration of, palliative, in home, and hospice services for life-limiting conditions other than cancer.

In this study, the majority of women (85 %) were hospitalised at least once during the observation period. This figure is slightly lower than that found in a recent 11-month Australian state-based study [11]. While not directly comparable, the findings are supported by other studies with similar healthcare systems who found that 60–79 % of older adults were admitted to hospital at least once in the last 6 months of life [9, 34]. Here, we were also able to show the compression of hospital use over the last year of life for six specific causes of death. Women who died of cancer had a median LOS of 23 days, with half admitted to hospital at least once within 5–12 months prior to death. Women who died of dementia/Alzheimer’s disease had a median LOS of 10 days and were admitted to hospital much closer to death. Although previous research has examined LOS of decedents over a 6 month period [18] or dichotomized diagnostic groups as ‘cancer vs non-cancer’ [11], the finding from our study is novel with LOS shown to be highly dependent on COD.

Glaser and colleagues first developed the concept of differing trajectories of dying, based on length and slope of functional decline [35]. Lunney et al. [19] analyzed these end of life trajectories for people with cancer, organ failure (congestive heart failure or chronic lung disease), and probable dementia (i.e. frailty) as well as those experiencing sudden death. Our findings, (extending this research in terms of hospital use and COD), are consistent with Lunney et al.’s regarding the trajectory for dementia. For this group of women, number of hospitalisations slowly increased across time and cumulative median LOS increased with proximity to death. Among women dying of cancer (when focused on hospital use), our findings contrast with the pattern of functional decline described by Lunney and colleagues [19], where these decedents maintain high levels of function until the last few months. Our data show that hospital use follows a more extended pattern over the final 12 months of life with a majority of women admitted 6–12 months prior to death, although we did see a large increase in admissions in the last 2 months. Our findings mirror that of Lunney and colleagues regarding organ failure, with hospital admissions extended over the 12 months and the primary reason for admission being chronic obstructive airway disorders. Our findings also show that in terms of median cumulative LOS, women who died of diabetes had similar patterns of hospitalisation as those who died of organ failure (particularly within the last 3 months of life). In the last month prior to death, more than half of women who died of either diabetes or organ failure had multiple hospital admissions. This is unsurprising given that heart failure is often a complication of diabetes, particularly for women [36]. For women who died of diabetes, approximately half of the reasons of admission in the last year were related to cardiovascular disease.

Moreover, while 7.0 % of women in this very old cohort died of diabetes, diabetes may become a more common COD in successive cohorts as its age-specific prevalence increases [37]. Diabetes management is therefore a health care priority, with self-management a key component of diabetes control [38, 39]. Best practice guidelines are primarily based on research involving adults aged ≤75 years [40] and have a strong emphasis on patient capacities to engage in self-management practices [41–43]. Yet, it is unknown how adults aged over 75 are able to self-manage diabetes in the community, particularly when they enter the final stages of life. Munshi et al. [44] found that barriers to self-care in older adults with poorly controlled diabetes related to a reluctance to make appropriate changes in insulin doses between clinic visits or during illness. The impact of intercurrent illness on the management of diabetes is particularly important, with effects on glucose metabolism through dysregulation of the hypothalamic-pituitary-adrenal and sympathetic-adrenal-medullary axes [45].

In our study, 17 % of women who died of diabetes had multiple hospital admissions within 6 months of death (and 40 % had multiple admissions within 2 months of death). It has been found that all-cause hospitalisation among adults with diabetes increased with insulin use, age, presence of chronic renal insufficiency, diagnosed hypoglycemia and a diabetes-related hospitalisation in the year prior [46].

Integrated healthcare pathways across the disease course and individualised care plans may assist in reducing unnecessary (and potentially unwanted) hospitalisations at the end of life. General practitioners are at the coal face of healthcare provision in Australia and may play an important role in not only coordinating and monitoring of care but also assisting with advanced care planning. Abarashi et al. [47] found that there was a particular need in primary care for integrated palliative care with optimal disease management for those with organ failure and that initiation of advanced care planning early in the chronic disease trajectory was imperative to enable patients to live as well as possible with progressive illness and die in line with their own preferences. Although further research is required among this age group, integrated community health care approaches have been shown to improve the care of those with diabetes (including a reduction in hospital admissions) and other chronic conditions [48, 49], while hospital in the home schemes have been suggested as one strategy to effectively manage respiratory exacerbations through either supported discharge after hospital admission or admission avoidance [50].

We also examined factors associated with a medium and palliative LOS (consistent with a LOS expected for palliative conditions whether or not the admissions were classified as palliative care). In contrast to De Korte-Verhoef et al. [13], women who died of cancer were most likely to have a palliative level LOS compared to other causes of death. The number of other medium level lengths of stay were associated with a 27 % increased risk of experiencing a palliative LOS while women who died in hospital or a nursing home were up to four times more likely to be admitted to hospital for a palliative LOS. Although the greatest proportion related to non-cancer diagnoses, a recent Western Australia-based study found that almost two-thirds of patients were in hospital in their final day of life (65 % of which were women) [11]. Interestingly, a small proportion of women did not have a hospitalisation at the end of life, with these women more likely to die of ‘other’ causes and have private health insurance. Whether this reflects issues surrounding access and equity to health services (including primary care) would require further exploration.

Taken together, the findings suggest that the Australian health system may have an inability to recognise and effectively manage end of life care [51]. Alternatively, health professionals may make conscience decisions to admit patients to acute care for long periods due to lack of access to appropriate palliative care and hospice services in community settings. Both state and federal governments in Australia have made a commitment to providing coordinated and consistent delivery of palliative care through the National Palliative Care Strategy (although mostly focused on cancer) [52]. The findings from this study indicate that improved access to, and knowledge of, palliative care services is required for older women who die of not only cancer but other life-limiting conditions. In our study, 49 % of women who died of organ failure and 55 % of women who died of cancer were admitted for a palliative LOS. It therefore begs the question: is the acute care setting the best place to administer end of life care for older women with these conditions? Can we do better to contribute to these women having a ‘good death’? The answers to these questions are extremely complex and require a shift in the collective consciousness about how we talk about death and dying and the importance of not interfering with the dying process with the instigation of futile treatments [53].

Importantly, studies suggest that hospitalisation at the end of life is often perceived as undesirable and can be associated with poorer outcomes for the patient and family [54–56]. It has been shown that up to 60 % of older adults die while in hospital [57]. This is despite research suggesting that the majority of people express a wish to die at home [58]. In our study, death in hospital ranged from 30 % (for dementia) to 59 % (for cancer and organ failure). A Swiss study found that age, female gender, multimorbidity and geographic location played a significant role in place of death for older patients [34], while a Dutch study examining the last 3 months of life found that people who received informal home care were more likely to die in hospital compared to institutional care [59]. It is argued that older adults who are in institutionalised care may have fewer transfers to hospital as they are already present in a ‘medicalised’ environment and therefore are having their needs met regarding treatment [47, 60]. This notion is also supported in countries with differing healthcare structures, such as the United States [21]. In our study however, 24 % of women with dementia experienced a medium level LOS and 35 % experienced a palliative LOS in their last year of life. Additionally, discharge to a nursing home was associated with a three-to-four fold increase in having an extended LOS.

It must be noted that although the majority of people express a wish to die at home, Gott et al. [61] found that this perception changed as death approached. As such, while death at home may be perceived as optimal, in actuality this may not be achievable. With the concept of ‘home’ a malleable concept within the end of life context [62], institutionalised forms of ‘home’ (i.e. hospice) may provide the best of both worlds. However, more research is required into the appropriateness and efficacy of such programs [63]. 

This study has a number of strengths including the longitudinal nature of the data, with older women being followed over a 12 year period. We were also able to link nationally representative self-report data to a large administrative dataset (involving both public and private hospitals) and the NDI in order to describe patterns of hospitalisation according to a number of causes of death for a community sample of women aged in their 70s through to their 80s. The study however has some limitations. Firstly, we classified women according to their primary COD. Comorbidities are common among older adults and potentially impacts on health service use and death trajectory. It is also important to acknowledge that patterns of hospital use at the end of life may have changed over the course of the observation period, including structural changes within the health care system as well as changes to health care provision as a result of improved technologies and new medications. Additionally, the self-report measures were taken from the last completed survey prior to death and as such considerable variability in the timing of measures may exist. However, we adjusted for this variation in the analyses. Also, although we examined palliative levels of LOS, we were unable to determine the type of care the participants were receiving (i.e. palliative or active life sustaining treatment). We also did not have information on other factors such as patient care preferences that may have also impacted on LOS. Further, this study focused solely on women. A Finnish study found significant differences in patterns of hospitalisation for men compared to women, with men having short lengths of stay [64]. Therefore, patterns of hospital use and predictors of hospital use in the last year of life may be markedly different for men.

## Conclusion

With global life expectancies increasing, effective planning of health services for future generations will require high quality information to understand how we may best provide appropriate care at the end of life. We were able to demonstrate the incremental increase in hospital use in the last 12 months of life, with different patterns of hospital use across six specific causes of death among older women. The solution to reducing acute care admissions at the end of life is extremely complex and requires a shift in perception regarding end of life care and chronic disease management.

## Additional files

Additional file 1: Table S1. Top 10 overall Australian Refined Diagnosis Related Groups (AR-DRG) based on principle diagnosis for all hospital admissions in the 12 months prior to death for women from the 1921–1926 cohort. (DOCX 16 kb)

## Abbreviations

ALSWH: Australian longitudinal study on women’s health
APDC: Admitted patients data collection
COD: Cause of death
GPs: General practitioners
LOS: Length of stay
NDI: National death index
NSW: New South Wales
