# Supplementary material for: End of life hospitalisations differ for older Australian women according to death trajectory: a longitudinal data linkage study
Source: BMC Health Serv Res. 2016 Sep 9;16(1):484. doi: 10.1186/s12913-016-1729-3 (PMC5017050; doi:10.1186/s12913-016-1729-3)
Supplement: Additional file 1: Table S1. — Top 10 overall Australian Refined Diagnosis Related Groups (AR-DRG) based on principle diagnosis for all hospital admissions in the 12 months prior to death for women from the 1921–1926 cohort. (DOCX 16 kb) [file 12913_2016_1729_MOESM1_ESM.docx]

**Table S1. Top 10 overall Australian Refined Diagnosis Related Groups (AR-DRG) based on principle diagnosis for all hospital admissions in the 12 months prior to death for women from the 1921-1926 cohort.**

| **AR-DRG Code** | **All**  **(%)** | **Other**  **(%)** | **Cancer**  **(%)** | **Organ failure**  **(%)** | **Dementia/ Alzheimer’s**  **(%)** | **Diabetes**  **(%)** | **Influenza**  **(%)** |
| --- | --- | --- | --- | --- | --- | --- | --- |
| Rehabilitation **(Z60)** | 10.8 | 12.0 | 7.6 | 12.6 | 11.6 | 9.9 | 15.7 |
| Stroke and other cerebrovascular disorders **(B70)** | 9.1 | 15.9 | 3.9 | 6.3 | 10.1 | 9.9 | 17.7 |
| Respiratory infection/ inflammatory **(E62)** | 8.3 | 4.6 | 6.5 | 10.0 | 9.4 | 6.2 | 33.3 |
| Heart failure and shock **(F62)** | 7.2 | 3.9 |  | 16.7 |  | 18.5 | 3.9 |
| Chronic obstructive airway disorder **(E65)** | 6.6 | 3.2 |  | 18.5 |  | 4.9 | 5.9 |
| Other factors influencing health status **(Z64)** | 5.7 | 6.0 | 4.2 | 5.2 | 7.3 | 6.2 | 13.7 |
| Circulatory disorders, AMI-invasive investigations **(F60)** | 5.2 | 6.0 |  | 6.7 | 3.6 | 18.5 |  |
| Other follow-up after surgical/medical care **(Z63)** | 4.9 | 5.0 | 6.3 | 3.3 | 2.9 | 7.4 |  |
| Other digestive system disorders **(G70)** | 4.7 | 3.9 | 7.3 |  | 3.6 | 4.9 |  |
| Arrhythmia, cardiac arrest and conduction disorders **(F76)** | 4.3 | 3.5 | 4.2 | 6.3 | 3.6 |  | 3.9 |
